# Supplementary material for: Stability and folding pathways of tetra-nucleosome from six-dimensional free energy surface
Source: Nat Commun. 2021 Feb 17;12:1091. doi: 10.1038/s41467-021-21377-z (PMC7889939; doi:10.1038/s41467-021-21377-z)
Supplement: Supplementary file 1 — Supplementary Information [file 41467_2021_21377_MOESM1_ESM.pdf]

Supporting Information for "Stability and folding pathways of tetra-nucleosome from six-dimensional free energy surface"

Xinqiang Ding,[†,‡] Xingcheng Lin,[†,‡] and Bin Zhang[*,†]

†*Departments of Chemistry, Massachusetts Institute of Technology, Cambridge, MA, USA*

‡*Contributed equally to this work*

E-mail: binz@mit.edu

Simulation Details

System setup

We built a complete structure for the tetra-nucleosome using the coordinates provided in the crystal structure (PDB ID:1ZBB).[1] Coordinates for histone proteins were swapped with those from the crystal structure with PDB ID 1KX5 to include histone tails. The DNA sequence was left unaltered, giving rise to linkers that are 20 bp long.

A total of three additional systems were built to study the impact of histone modifications on chromatin organization. The first system mimics H4K16 acetylation by removing the positive charge on the 16th Lysine residue of histone H4 from all nucleosomes. To account for a possible change in the secondary structure as a result of acetylation, the second system biases all the histone H4 N-terminal tails into a beta-sheet conformation with the following potential

$$U_b = \frac{k}{2}(Q_t - 1)^2, \quad Q_t = \frac{1}{N}\sum_{i=1}^{24}\sum_{j=i+3}^{24} \exp\left[-\frac{(r_{ij} - r_{ij}^o)^2}{2\sigma^2}\right]. \tag{S1}$$

We used $k = 5000$ kJ/mol and $\sigma = 0.05$ nm. N is the total number of amino acid pairs and r_{ij}^o is the distance between reside i and j in the β-sheet structure of the histone tail seen in all-atom simulations.[2] Finally, we studied another system that mimics the acetylation of the entire H4 tail by setting the positive charges on residues 3,5,8,12,16,17,19, 20 and 23 to zero and without the secondary structure bias.

Force field setup

We combined the 3SPN.2C DNA model[3] and the structure-based Cα model[4,5] to create a coarse-grained force field for modeling protein-DNA complexes. We represent each DNA base with three beads and every amino acid with one bead. The energy function of the system includes contributions from intra-DNA, intra-protein, inter-protein, and protein-DNA interactions. Parameters from 3SPN.2C were directly applied to model intra-DNA interac-

tions for the tetra-nucleosome sequence studied here. When simulating proteins with the structure-based model, we treated each histone octamer as a single unit. Intra-protein interactions, therefore, refer to all interactions within an octamer, while inter-protein interactions correspond to those between octamers. To ensure the stability of the histone octamer during simulation, we included a list of native contacts for intra-protein interactions. These contacts were generated from the PDB structure (ID: 1KX5) using the Shadow contact map.[6] Two residues were considered in contact if their minimal atomic distance is 6 Å or less, regardless of whether they are from the same protein chain or not. We scaled the energy of the structure-based model by a factor of 2.5 to 0.6 kcal/mol to prevent the protein complex from unfolding at a temperature of 300 K. Detailed expressions of the energy function for the protein and DNA models can be found in Ref. 3 and 7. Electrostatic interactions modeled at the Debye-Hückel level were included between charged beads, including DNA phosphates, LYS, ARG, GLU, and ASP residues. A salt concentration of 150mM was used for the screening effect. In addition, a weak, non-specific Lennard-Jones potential was applied between all protein-DNA beads. Detailed expression for these potentials can be found in Ref. 8.

To estimate the impact of protein-protein interactions on tetra-nucleosome stability, we modeled non-bonded interactions between amino acids I and J using the Lennard-Jones potential.

$$U_{\text{LJ}}(r) = \begin{cases} 4\epsilon_{IJ}\left[\left(\frac{\sigma}{r}\right)^{12} - \left(\frac{\sigma}{r}\right)^{6}\right] - E_{\text{cut}} & r < r_c \\ 0 & r \geq r_c \end{cases} \tag{S2}$$

with $\sigma = 5.0$Å $r_c = 2.5\sigma$ and $E_{\text{cut}} = 4\epsilon_{IJ}[(\sigma/r_c)^{12} - (\sigma/r_c)^6]$. The contact matrix ϵ_{IJ} was parameterized using the Miyazwa-Jernigan (MJ) potential scaled by a factor 0.4. The scaling factor was chosen such that the potential provides a balanced prediction of protein size for both ordered and disordered proteins.

Definition of collective variables Q and R_g

As defined in the main text, Q measures the similarity of a given tetra-nuclesome configuration to the crystal structure and is defined as

$$Q = \frac{1}{6}\sum_{i=1}^{3}\sum_{j=i+1}^{4} \exp\left[-\frac{(r_{ij}-r_{ij}^{o})^2}{2\sigma^2}\right], \tag{S3}$$

where r_{ij} measures the distance between the center of the two nucleosomes. During the simulations, not all atoms were included to compute the center for efficiency. Instead, we only include every other 3 residues from the rigid part of the histone proteins. The list of residues included is provided below.

```
45,48,51,54,57,60,63,66,69,72,75,78,81,84,87,90,93,96,99,102,105,108,111,
114,117,120,123,126,129,132,135,162,165,168,171,174,177,180,183,186,189,
192,195,198,201,204,207,210,213,216,219,222,225,228,231,234,237,258,261,
264,267,270,273,276,279,282,285,288,291,294,297,300,303,306,309,312,315,
318,321,324,327,330,333,336,339,342,345,348,351,402,405,408,411,414,417,
420,423,426,429,432,435,438,441,444,447,450,453,456,459,462,465,468,471,
474,477,480,483,486,531,534,537,540,543,546,549,552,555,558,561,564,567,
570,573,576,579,582,585,588,591,594,597,600,603,606,609,612,615,618,621,
648,651,654,657,660,663,666,669,672,675,678,681,684,687,690,693,696,699,
702,705,708,711,714,717,720,723,747,750,753,756,759,762,765,768,771,774,
777,780,783,786,789,792,795,798,801,804,807,810,813,816,819,822,825,828,
831,834,837,888,891,894,897,900,903,906,909,912,915,918,921,924,927,930,
933,936,939,942,945,948,951,954,957,960,963,966,969,972
```

The radius of gyration, R_g, is computed as

$$R_g = \sqrt{\frac{1}{4}\sum_{i=1}^{4}(\mathbf{r}_i - \mathbf{r}_{\mathrm{com}})^2}, \tag{S4}$$

where $\mathbf{r}_i$ is the geometric center of the i-th nucleosome using the coordinates of residues listed above. $\mathbf{r}_{\text{com}}$ is the center of mass coordinate for all nucleosomes.

Rigid body treatment

To improve computational efficiency, we modeled the core region of each nucleosome as rigid bodies. This region includes the folded segments of the histone octamer and the central 73 base pair DNA. The rigid units consist of six degrees of freedom for translation and rotation, and all atoms within the same unit move concurrently. Rigidification of inner nucleosome core removes fast degrees of freedom and allows the use of a larger time step in simulations. Computational cost is reduced as well since interactions within the rigid bodies are no longer evaluated. Our setup maintains the flexibility of the linker DNA, the outer layer nucleosome DNA, and disordered histone tails. Since the inner DNA layer is known to bind tightly to the well-folded histone core in resting nucleosomes, we anticipate the rigid body treatment to be a good approximation.

To probe the impact of the rigid body treatment on the model's accuracy, we carried out two long-timescale simulations to determine the statistical distribution of chromatin conformations with and without rigid body treatment. The two simulations both lasted for 150 ns with a time step of 10 and 2 fs, respectively. We restricted the tetra-nucleosome to configurations close to the crystal structure via the harmonic potential $\frac{k}{2}(Q-0.8)^2$ with $k = 10000$ kJ/mol. This restraint does not affect conclusions on the accuracy of the rigid body dynamics and helps reduce the computational cost needed for statistical convergence. Though interesting, configurations deviating from the crystal structure are not necessary for validating the accuracy of the rigid body dynamics. If included, they will introduce structural rearrangements occurring at a slow timescale to hinder the convergence of ensemble averages.

As shown in Fig. S11b, the free energy profiles computed for the six inter-nucleosome distances from the two independent simulations are all within errorbars. Since the rigid body treatment does not impact the statistics of these distances, it will not affect the accuracy of

the mean force estimations and the free energy surface computed from the forces. Therefore, we anticipate that the main text's conclusions will not be affected by the rigid body treatment.

Driving chromatin folding with advanced sampling techniques

We combined metadynamics together with temperature accelerated molecular dynamics (TAMD) to bias the simulations along two collective variables R_g and Q. TAMD introduces two fictitious variables $r(t)$ and $q(t)$ coupled with R_g and Q via the following harmonic potential

$$V = \frac{k_r}{2}(R_g(\mathbf{r}) - r(t))^2 + \frac{k_q}{2}(Q(\mathbf{r}) - q(t))^2, \tag{S5}$$

with $k_r = 200$ kJ/mol/nm^2 and $k_q = 10000$ kJ/mol. $\mathbf{r}$ represents Cartesian coordinates of the tetra-nucleosome. To further enhance conformational sampling, we incorporated metadynamics biasing for the fictitious variables with a time-dependent potential

$$V_{\mathrm{m}}(t) = \sum_{l,t_l<t} h\left[e^{-(r(t)-r(t_l))^2/2\sigma_r^2} + e^{-(q(t)-q(t_l))^2/2\sigma_q^2}\right]. \tag{S6}$$

The potential was deposited at every $l = 500$ steps with a barrier height $h = 0.1$ kJ/mol, and approximated using a two-dimensional grid with spacing of 0.2 nm and 0.01 for computational efficiency. $\sigma_r = 0.5$nm and $\sigma_q = 0.02$. Langevin dynamics was used to evolve the fictitious variables along with the system's equation of motion

$$\begin{cases} \mu_r \ddot{r}(t) &= -\nabla_r(V + V_{\mathrm{m}}) - \mu_r \gamma \dot{r}(t) + \eta_r(t) \\ \mu_q \ddot{q}(t) &= -\nabla_q(V + V_{\mathrm{m}}) - \mu_q \gamma \dot{q}(t) + \eta_q(t). \end{cases} \tag{S7}$$

The masses $\mu_{r/q} = k_{r/q}(\frac{\tau}{2\pi})^2$, with $\tau = 10$ ps. The friction coefficient $\gamma = 10$ ps^{-1}. The random forces were defined as $\eta_{r/q}(t) = \sqrt{2\mu_{r/q}\gamma k_B T_c}$. The effective temperature for these variables does not need to follow that of the real system and we used $T_c = 1000K$ to accelerate

the dynamics at high temperature and facilitate the transition across barriers.

Computing free energy surface from mean forces with neural networks

To estimate mean forces at the 10000 selected centers, we carried out restrained molecular dynamics simulations with the harmonic biasing potential

$$V_b = \frac{1}{2}\sum_{i=1}^{3}\sum_{j=i+1}^{4} k(d_{ij}(\mathbf{r}) - d^o_{ij})^2, \tag{S8}$$

where i and j are indexes of the four nucleosomes, $k = 1000$ kJ/mol/nm^2, and d^o_{ij} is the inter-nucleosome distances between nucleosome i and j for the selected center $\mathbf{d_o}$. The mean forces were estimated as

$$\mathbf{F}^o_{ij} = \frac{1}{T}\sum_{t=1}^{T} k(d^t_{ij} - d^o_{ij}). \tag{S9}$$

Here $T = 50,000$ represents the number of configurations collected from a 500,000 step long trajectory.

To make the neural network's output invariant with respect to nucleosome indexing order, i.e., $A(\mathbf{d} = (d_{12}, d_{13}, d_{14}, d_{23}, d_{24}, d_{34})) = A(\tilde{\mathbf{d}} = (d_{34}, d_{24}, d_{14}, d_{23}, d_{13}, d_{12}))$, the distances are converted into symmetrical features $\mathbf{s}(\mathbf{d}) = (s_1(\mathbf{d}), s_2(\mathbf{d}), s_3(\mathbf{d}), s_4(\mathbf{d}), s_5(\mathbf{d}), s_6(\mathbf{d}))$ as follows:

$$\begin{aligned}
s_1 &= d_{12} + d_{34}\\
s_2 &= d_{13} + d_{24}\\
s_3 &= d_{14}\\
s_4 &= d_{23}\\
s_5 &= d_{12}\cdot d_{13} + d_{24}\cdot d_{34}\\
s_6 &= d_{12}\cdot d_{13}^2 + d_{24}^2\cdot d_{34}.
\end{aligned} \tag{S10}$$

From the above definition, it is straightforward to verify that $\mathbf{s}(\mathbf{d}) = \mathbf{s}(\tilde{\mathbf{d}})$. In addition, given any $\mathbf{s}$ in the range of $\mathbf{s}(\mathbf{d})$, two solutions of $\mathbf{d}$ exist for Eq. (S10) and these two solutions corresponds to the two different ways of indexing nucleosome. Specifically, if one of the solution is $\mathbf{d} = (d_{12}, d_{13}, d_{14}, d_{23}, d_{24}, d_{34})$, the other solution will be $\tilde{\mathbf{d}} = (d_{34}, d_{24}, d_{14}, d_{23}, d_{13}, d_{12})$. Therefore, the features $\mathbf{s}(\mathbf{d})$ are symmetric and only symmetric to the two ways of indexing nucleosomes.

Using the symmetric features as input, i.e., $A(\mathbf{d}) = A(\mathbf{s}(\mathbf{d}))$, a neural network with two fully connected hidden layers, each of which has 200 nodes, was used to parameterize the free energy. The neural network was trained by minimizing the loss function

$$||(-\nabla A(\mathbf{d})) - \mathbf{F}(\mathbf{d})||^2 + \lambda ||\mathbf{w}||^2, \tag{S11}$$

where $\mathbf{w}$ are weight parameters of the neural network. λ is the weight decay factor and acts as a regularizer of optimization. Overall, the neural network has 41801 ($7 \times 200 + 201 \times 200 + 201 \times 1 = 41801$) parameters, which is smaller than the total number of constraints $10000 \times 6 = 60000$. The Adam optimizer[9] was used to train the neural network for 100000 steps with a learning rate of 0.001. We observed that as λ increases from 10^{-5} to 10^{-4}, 6×10^{-4}, and 10^{-3}, the correlation between forces computed using the model and the training data decreases but the model become more robust and reproducible (Fig. S3). We, therefore, computed all results presented in main text with $\lambda = 6 \times 10^{-4}$, though the free energy profiles computed using different values of λ are comparable (Fig. S4). To further prevent over fitting and improve the robustness of neural networks, we trained 30 models independently and used the average results to estimate the final free energy.

Free energy as a function of d_{13} calculated from umbrella sampling

To validate the accuracy of the free energy surface computed via neural network, we carried out additional simulations to determine the one-dimensional free energy profile as a function

of distance between nucleosome 1 and 3, d_{13}.

A total of 13 umbrella windows was used to calculate the free energy. In these simulations, the distance d_{13} was biased towards values of $d_{13}^o = \{5,8,9,10,13,15,20,24,25,8.5,12.5,17.5,22.5\}$ nm with harmonic potentials $k/2 \cdot (d_{13} - d_{13}^o)^2$, respectively. $k = 5$ kJ/mol/nm^2, except for umbrellas windows at $d_{13}^o = \{6.5, 8.5, 13\}$ nm ($k = 10$ kJ/mol/nm^2) and at $d_{13}^o = 8$ nm ($k = 40$ kJ/mol/nm^2). All trajectories were initialized from the the crystal structure and the total simulation lengths are provided in Table S1. Tetra-nucleosome configurations were collected at every 1000 steps and the final data set was evenly broken into five blocks. The first two blocks were discarded as equilibration and the errorbars shown in Fig. 3c of the main text were estimated using free energy profiles determined from the last three blocks.

Details on finite temperature string method calculations

The finite temperature string (FTS) method[10] was used to search for folding pathways that connect an extended configuration with inter-nucleosome distances $\mathbf{d}_e = (15, 25, 35, 15, 25, 15)$ nm to a stacked conformation with inter-nucleosome distances $\mathbf{d}_e =$ (13.66, 6.35, 11.86, 13.65, 6.71, 11.12) nm.

To thoroughly explore folding pathways on the six dimensional free energy surface, we performed multiple repeats of FTS with different initial strings chosen as follows (see Fig. S6a). These strings consist a total of 15 beads, with $\mathbf{d}_1 = \mathbf{d}_e$ and $\mathbf{d}_{15} = \mathbf{d}_c$. The inter-nucleosome distances (d_{13}, d_{24}) of the middle bead (the 8th bead) $\mathbf{d}_8$ was chosen from the list $\{(10.0, 25.0)$, $(12.5, 22.5)$, $(15.0, 20.0)$, $(17.5, 17.5)$, $(20.0, 15.0)$, $(22.5, 12.5)$, $(25.0, 10.0)\}$. For beads 2 to 7, their d_{13} and d_{24} were determined via a linear interpolation between the first and the middle bead. The corresponding values for beads from 9 to 14 were similarly determined via a linear interpolation between the middle and the last bead. For beads from 2 to 14, the rest of the inter-nucleosome distances $(d_{12}, d_{14}, d_{23}, d_{34})$ were chosen randomly under the constraint that beads with higher index have larger coordinate values. These initial strings cover both sequential and concerted pathways by design.

Starting from their initial values, strings were optimized using the algorithm outlined in Ref. 10. Both the first and the last bead were fixed throughout the optimization. Numerical integration of the Langevin dynamics with a time step of $\Delta t = 0.001$ was performed and used to update the running average of inter-nucleosome distances for configurations sampled within each one of the 13 Voronoi cells. Mean inter-nucleosome distances from each cell were then used to update the string with a step size $\Delta\tau = 0.1$. The parameter κ controlling the smoothness of the string was set as 0.1. Strings with 15 beads were optimized for 5000 steps such that the root mean square derivation of the path between two consecutive steps is less than 0.001. Afterwards, strings were reparameterized with 31 beads by interpolation to provide a finer characterization of the folding pathway. An additional 10000 steps of optimization were carried out to obtain the final results.

Low-dimensional projection of free energy surfaces

To project the six-dimensional free energy profiles learned using the neural network into lower dimensions, we performed Markov chain Monte Carlo (MCMC) sampling with temperature replica exchange in the six-dimensional distance space. Specifically, 50 Markov chains at different temperatures were used. The temperature of chain 1 is $T_1 = 300$ K, which is the same as that used in the coarse-grained simulations. The temperature of chain m is $T_m = 300/\alpha_m$ K, where $\alpha_m = 1 - 0.99 \times (m-1)/49$, and $m = 2, ..., 50$. All the Markov chains used the distances from the tetra-nucleosome crystal structure, which are $(d_{12}, d_{13}, d_{14}, d_{23}, d_{24}, d_{34}) = (15.42, 5.81, 15.15, 14.90, 5.82, 15.43)$nm, as their initial state. At each step of chain m, a candidate state was generated by sampling from a six-dimensional Gaussian distribution $\mathcal{N}(\mu, \sigma_m^2\mathbf{I})$, where μ is the state at the current step and σ_m^2 is the variance of the proposal distribution. Then the candidate state is accepted or rejected based on the Metropolis criteria at the corresponding temperature T_m. To make the MCMC sampling efficient, the standard variation, σ_m, of the proposal distribution was set based on the ruggedness of the free energy landscape at the temperature T_m as follows. Initially, σ_m was

set to $\sigma_m = 2.0 - 1.9 \times (m-1)/49$ for $m = 1, ..., 50$. Then in the initial 1×10^5 steps of burn-in sampling, the value of σ_m was dynamically changed within the range from 0 to 3 such that the accumulative acceptance rate in the chain m is between 0.4 and 0.6, i.e., if the accumulative acceptance rate is lower than 0.4 or higher than 0.6, the value of σ_m is decreased or increased by 2%, respectively. After the initial 1×10^5 steps of burn-in sampling, the values of σ_m were fixed in the following production sampling. 1×10^7 steps of production sampling were conducted and samples were saved for every 100 steps. Samples between nearest neighbour chains were attempted to exchange every 10 steps and the exchange is accepted or rejected based on Metropolis criteria. Samples collected from all the 50 Markov chains were re-weighted using the multiple state Bennett acceptance ratio (MBAR) method.[11,12] All the re-weighted samples were used to construct free energy profiles in lower dimensional distance spaces such as d_{13} and $d_{13} - d_{24}$ using histograms.

Simulation details of dinucleosomes with different linker lengths

To further evaluate the near-atomistic model's accuracy, we simulated two dinucleosomes with linker DNAs of 50 and 55 base pairs in length. These two systems were characterized by van Noort and coworkers using single pair Förster resonance energy transfer (spFRET) experiments.[13] The initial model was constructed by connecting two nucleosomes extracted from the tetranucleosome structure (PDB ID: 1ZBB) with a non-repeating linker DNA (TATGACAGTGCATCACGGGGTGTGACAGTGCATCACGGGGTGAGATCGCTA and TATGACAGTATCGAGCATCACGGGGTGAGATCGCTTATGACAGTATCGAGCATCAA) using the 3DNA package.[14] We then replaced the nucleosomal DNA with the 601-sequence from PDB 3LZ1,[15] and swapped coordinates of histone proteins with those from the PDB 1KX5 to include histone tails. Finally, an extra 12 base-pair linker DNA (sequence: TATGACAGTGCA) was added to the free end of each nucleosome to reproduce the same system studied by van Noort and coworkers.[13]

The simulations were carried out with the same setup as in the tetranucleosome system.

Parallel tempering at temperatures ranging from 300K to 410K with a spacing of 10K was used to enhance conformational sampling. Each replica lasted for 21 million steps, and exchanges between replicas were attempted at every 100 steps. We used the last 11 million steps of simulation data at 300 K to construct the probability density distributions (Fig. S10).

To study nucleosomal DNA unwrapping, we monitored the distance between the two DNA base pairs where the experimental donor and acceptor were attached.[13] They correspond to the second base pair from the nucleosome exit and the 10th base pair from the dyad. The positions of solvent exposed phosphate bead of each base pair were used to compute the distance. As shown in Fig. S10a, the most probable distance from simulations is 4.31 nm, and the corresponding experimental value is 5.09 nm. Given the inclusion of fluorophores, typically 1 nm in size, in the experimental but not simulated distance, the agreement between the two is acceptable. The experimental curves were converted from FRET efficiency figure (Fig. 7(a) of Ref. 13) using the relation between distance r and FRET efficiency E: $r = R_0(1/E - 1)^{\frac{1}{6}}$, where the Förster distance R_0 of the experimentally used fluorophores was set to 5.5 nm.[16]

Both the simulated and experimental distance probability distributions exhibit long tails at large values, indicating the presence of unwrapped configurations. To further quantify the population of the unwrapped nucleosomes, we converted the distance r from our simulation to the FRET efficiency E using the formula: $E = 1/(1+(\frac{r+r_0}{R_0})^6)$, where r_0 is the approximate size of the fluorophore. Following van Noort et al.,[13] we further defined the unwrapped state as the configurations with FRET efficiency less than 0.3. Considering $r_0 \approx 1.0$ nm, the population of the unwrapped state was estimated as 19% and 9% for the di-nucleosomes 55 and 55 bp long linker DNA. These values are comparable to the experimental results of 22% and 12%.

Explicit solvent simulations of the tetra-nucleosome

The simulations performed in the main text using the near-atomistic model suggest that the stacked configurations shown in Fig. 2 are more favorable than the PDB structure. To further validate this prediction, we carried out additional tetra-nucleosome simulations using an independent force field, SIRAH.[17] While molecules are still treated at a coarse-grained level, this force field includes explicit representations of water molecules and ions and is expected to describe the solvation effect and electrostatic interactions accurately. SIRAH has been used to study membrane proteins,[18] protein-peptide recognition,[19] and protein-DNA binding interfaces.[20]

To construct the initial configuration of these simulations, we solvated the PDB structure for the tetra-nucleosome[1] using coarse-grained water molecules with a octahedron box. The size of the simulation box was chosen to ensure a minimal 2.0 nm distance of any tetranucleosome atoms from the box edge. Monovalent ions of NaCl were added to reach an ionic concentration of 150 mM. The final system consists of 335,906 particles. Starting from the initial configuration, we performed constant temperature of 300 K and pressure of one bar (NPT) simulations using the velocity-rescaling based thermostat,[21] and the Parrinello-Rahman barostat.[22] Electrostatic interactions were treated using the Fast smooth Particle-Mesh Ewald (SPME) method.[23] All simulations were performed using Gromacs[24] with a time step of 20 fs.

We first carried out an unbiased 4-μs-long trajectory. To quantify the rotation between the two columns of nucleosomes, we monitored the angle formed between the 1-3 and the 2-4 nucleosome vectors (Fig. S5a). The two vectors were defined using the geometric centers of the non-tail part of the histone octamer from each nucleosome. As shown in Fig. S5b, the angle continuously decreases from 111 to 80 degree along the trajectory, supporting the stability of the more aligned configurations shown in Fig. 2 of the main text.

To ensure the statistical significance of the above observation from a dynamical trajectory, we further computed the free energy profile for the rotational angle using umbrella sampling

simulations. Details regarding the umbrella centers, the spring constants for biasing, and the total lengths of the simulations are provided in Table S2. To speed up the equilibration of the system into the reference angle, we applied a large spring constant (20000 kcal/(mol·degree^2)) in the first 30 ns of each trajectory, and gradually relaxed the constant to the values shown in Table S2 in the following 10 ns simulation. The first 100 ns of each trajectory was discarded for free energy calculation.

As shown in Fig. S5c, the calculated free energy profile strongly favors configurations with small rotational angles that resemble the structures presented in Fig. 2. Therefore, the simulations performed using the SIRAH force field supports the accuracy of the near-atomistic model used in the main text for predicting chromatin organization.

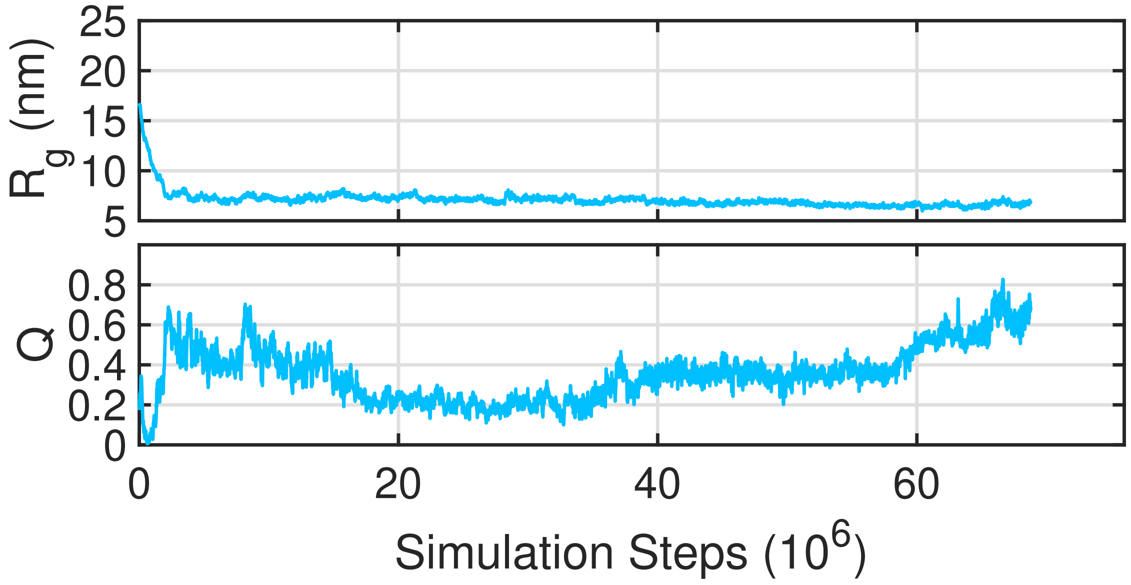

Figure S1: Time evolution of the radius of gyration (top) and the fraction of native contacts (bottom) along a trajectory without biasing from meta-dynamics or TAMD. Initial structure of this simulation is identical to that shown in Fig. 1a of the main text.

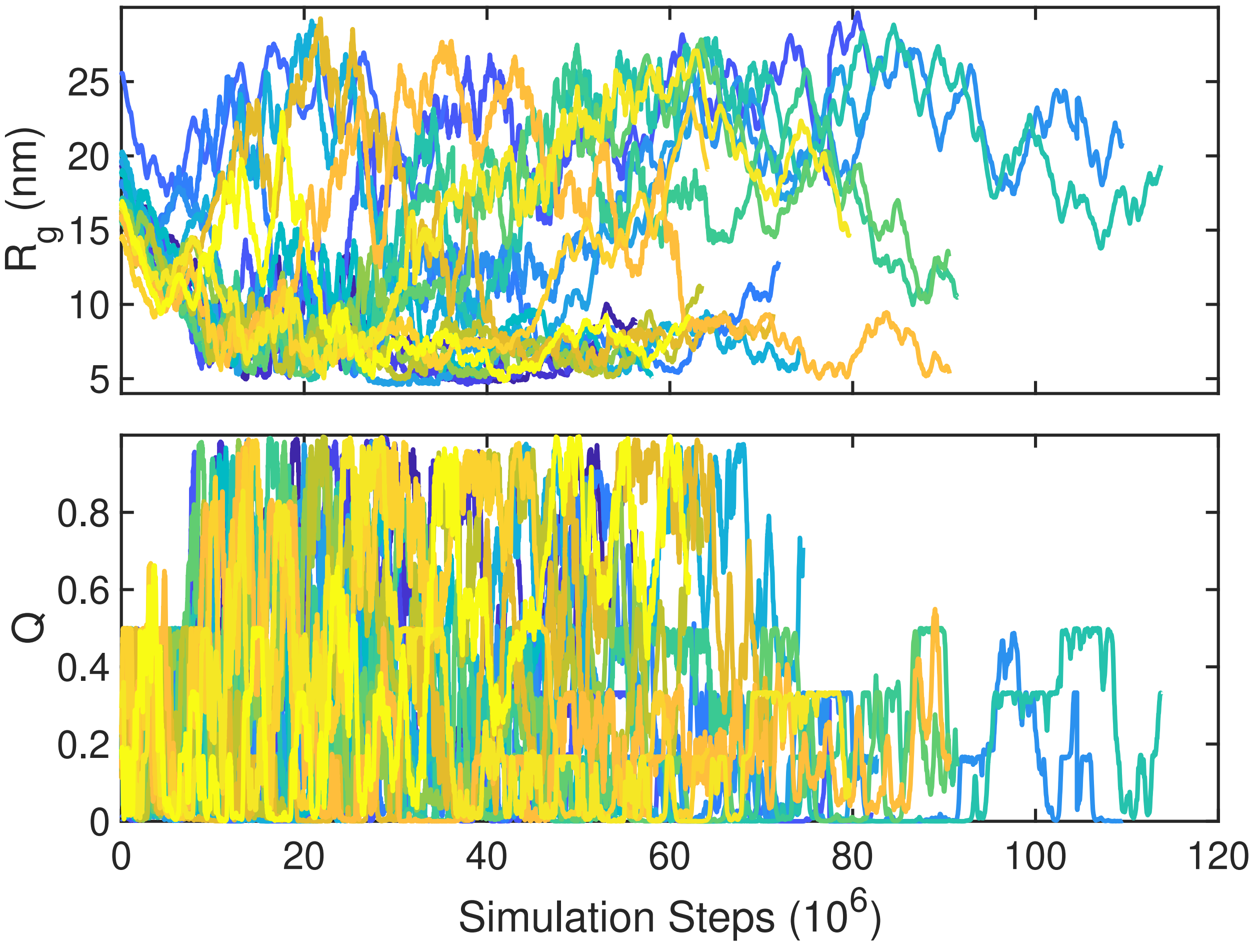

Figure S2: Time evolution of the radius of gyration (top) and the fraction of native contacts (bottom) along the 20 trajectories that combine meta-dynamics with temperature accelerated molecular dynamics. See text *Driving chromatin folding with advanced sampling techniques* for simulation details.

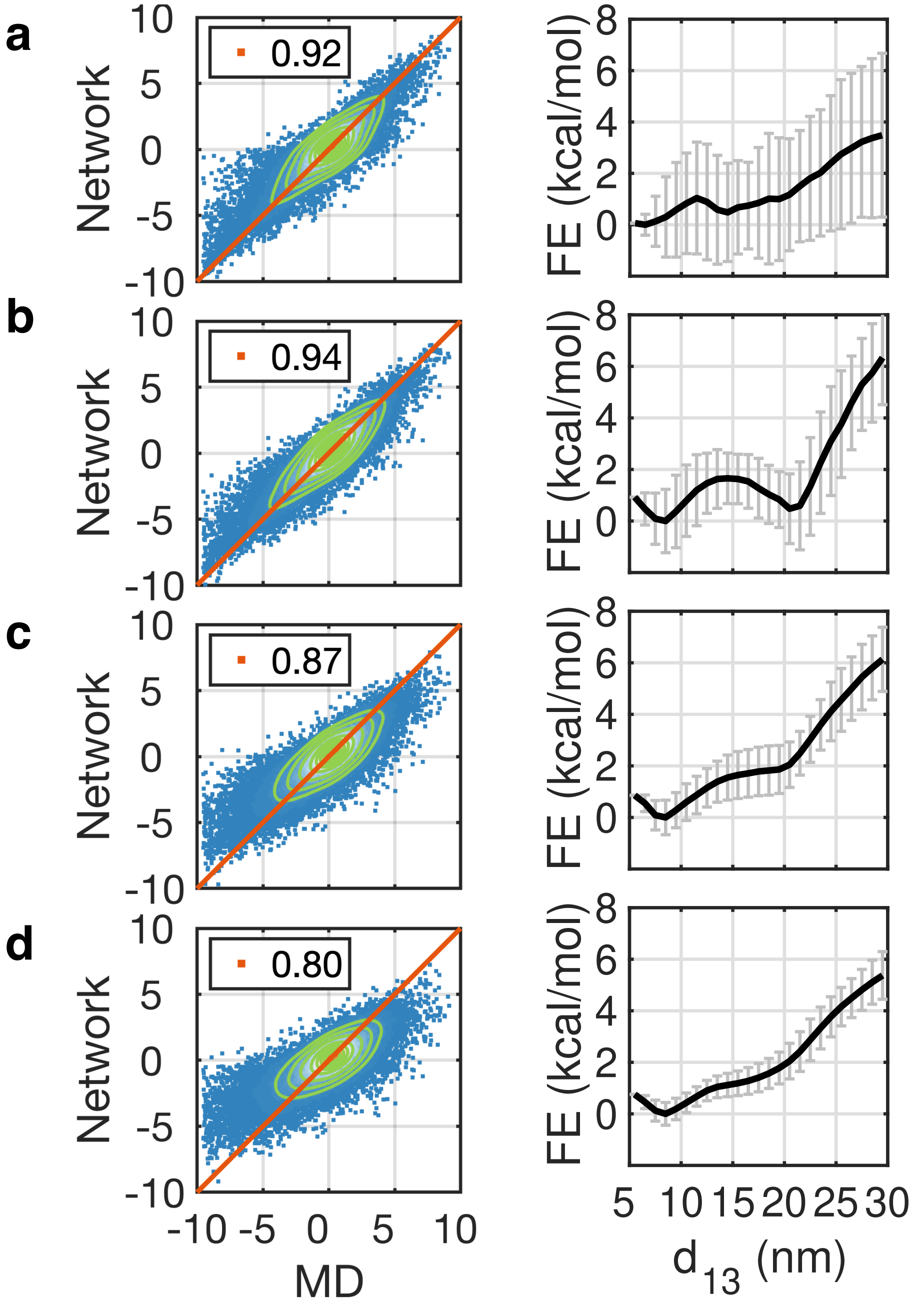

Figure S3: **Dependence of neural network performance on the strength of regularization λ.** (*Left*) Comparison between mean forces in the unit of kcal/mol/nm determined from restrained molecular dynamics (MD) simulations and neural network prediction for $\lambda = 10^{-5}$ (a), $\lambda = 10^{-4}$ (b), $\lambda = 6 \times 10^{-4}$ (c), and $\lambda = 10^{-3}$ (d). Pearson correlation coefficients are provided in the legends. The density of data points increases as the color changes from blue to white to red. The green lines are constant density contours and the red diagonal line is shown as a guide for the eye. (*Right*) The resulting free energy profile as a function of 1-3 nucleosome distance for different values of λ. Error bars correspond to standard deviations calculated from 30 independent network optimizations. See text *Computing free energy surface from mean forces with neural network* for calculation details.

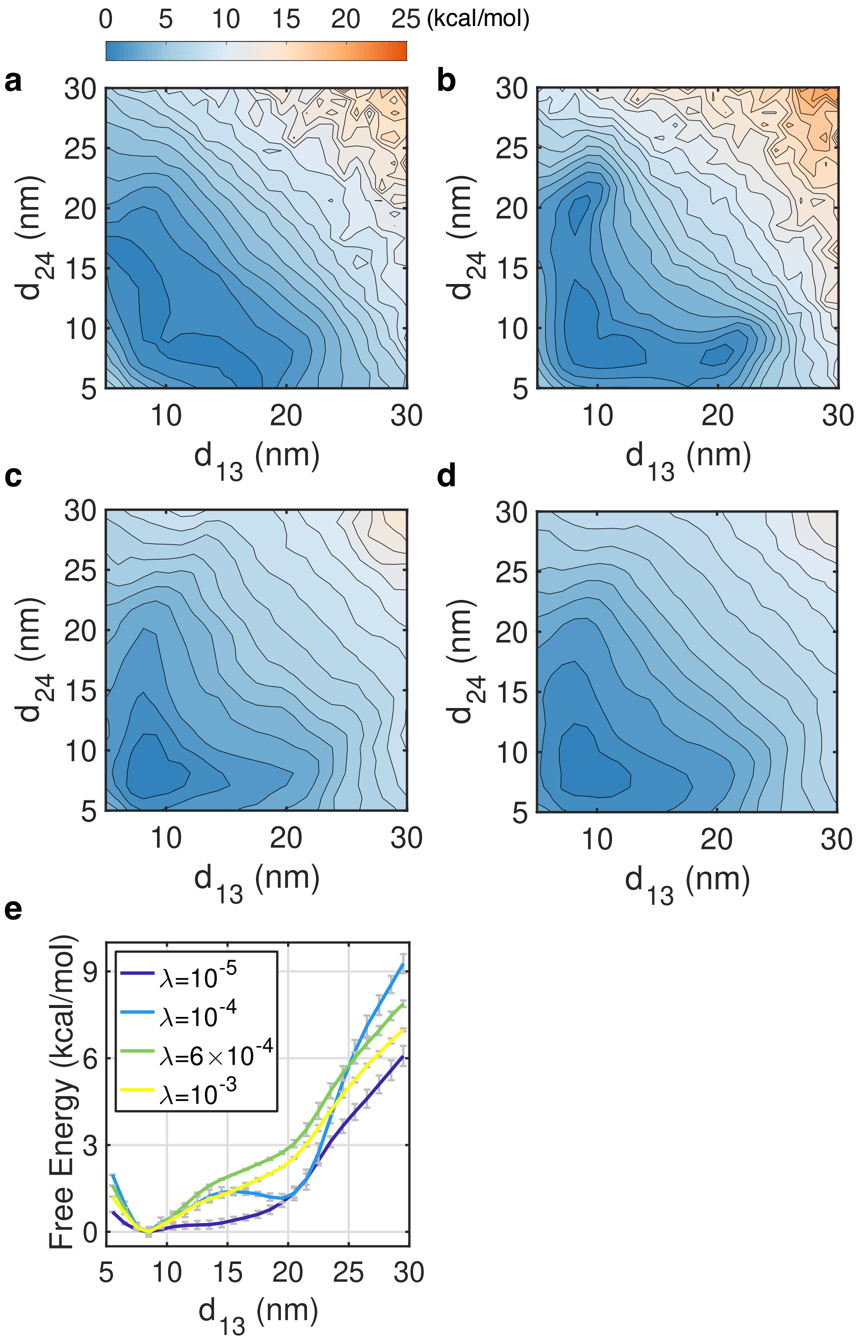

Figure S4: **Impact of network regularization on the final free energy profile.** (a,b,c, d) Contour of two dimensional free energy profile as a function of the distance between 1-3 (d_{13}) and 2-4 (d_{24}) nucleosomes for $\lambda = 10^{-5}$ (a), 10^{-4} (b), 6×10^{-4} (c) and 10^{-3} (d). (e) Comparison of the corresponding free energy profiles as a function of 1-3 nucleosome distance. Error bars correspond to standard deviations calculated from the average of three different ensembles, each one of which consists of 30 independent network optimizations. See text *Computing free energy surface from mean forces with neural network* for calculation details.

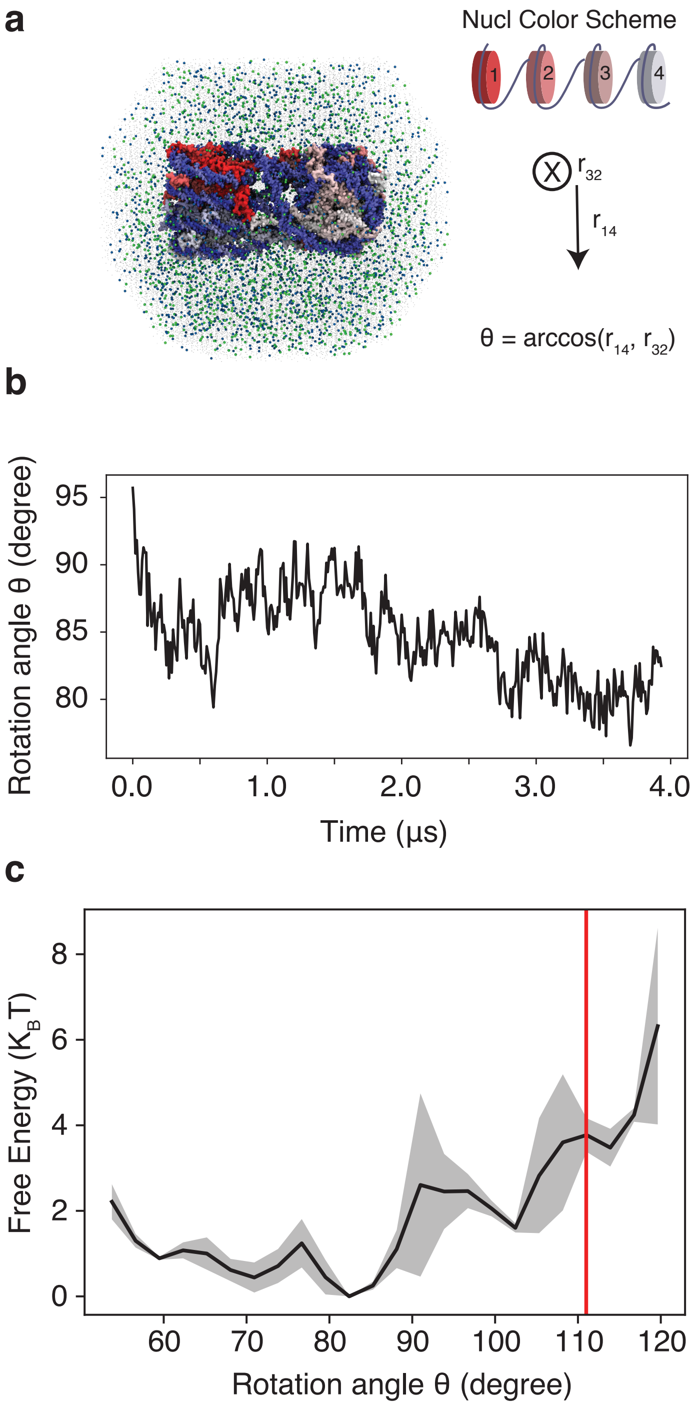

Figure S5: **Explicit solvent simulations of the tetranucleosome system with the SIRAH force field supports the stability of more aligned stacked configurations presented in Fig. 2 of the main text.** (a) Illustration of the initial configuration used for explicit solvent simulation and the definition of the rotation angle, θ. (b) Time evolution of the angle θ between the two nucleosome columns along a 4 μs-long trajectory. (c) The free energy profile as a function of the rotation angle favors more aligned configurations with smaller angle than the PDB structure (red). The shaded regions correspond to standard deviation of the mean calculated with block averaging. See text *Section: Explicit solvent simulations of the tetra-nucleosome* for more discussions.

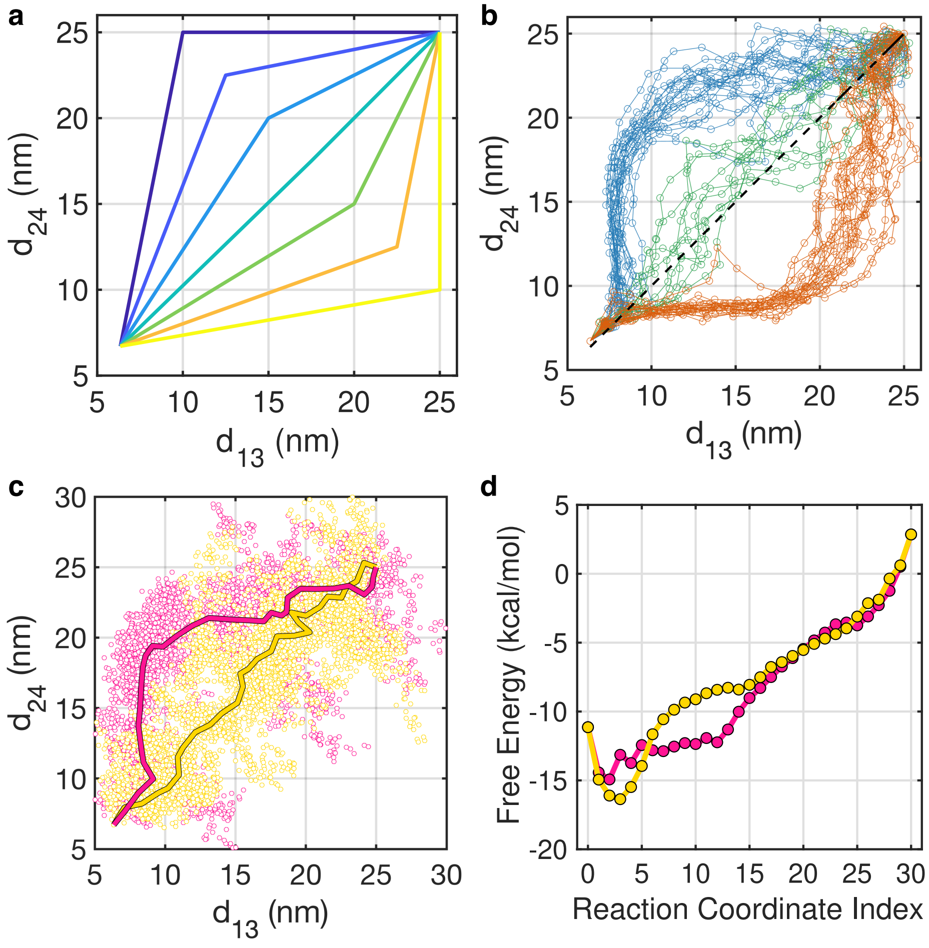

Figure S6: **Chromatin folding pathways determined using the finite temperature string method.** (a) Configurations along the paths used to initialize the string method. As explained in the text *Details on finite temperature string method calculations*, we first generated the seven paths shown in the plot using the coordinates of d_{13} and d_{24}. Each path was further used to produce ten paths that differ in the other inter-nucleosome distances via random sampling. (b) Final paths obtained from string method calculations . Only 49 out of the 70 were shown for clarify. The remaining paths appear to bridge between the transition tubes, possibly due to unrealistic initializations. This plot supports the presence of both concerted and sequential pathways. The fluctuation among paths within each transition tube arises from both the ruggedness of the free energy surface and the stochastic nature of the string method algorithm. (c) The sequential (pink) and concerted (yellow) pathways plotted over the two inter-nucleosome distances. The dots correspond to tetra-nucleosome configurations obtained from individual simulations restricted to Voronoi cells defined using images along the path. They represent fluctuations around the path on the scale of $k_B T$. The concerted pathway is identical to the one shown in Fig. 4 of the main text. (d) Free energy profiles along the two pathways shown in part c.

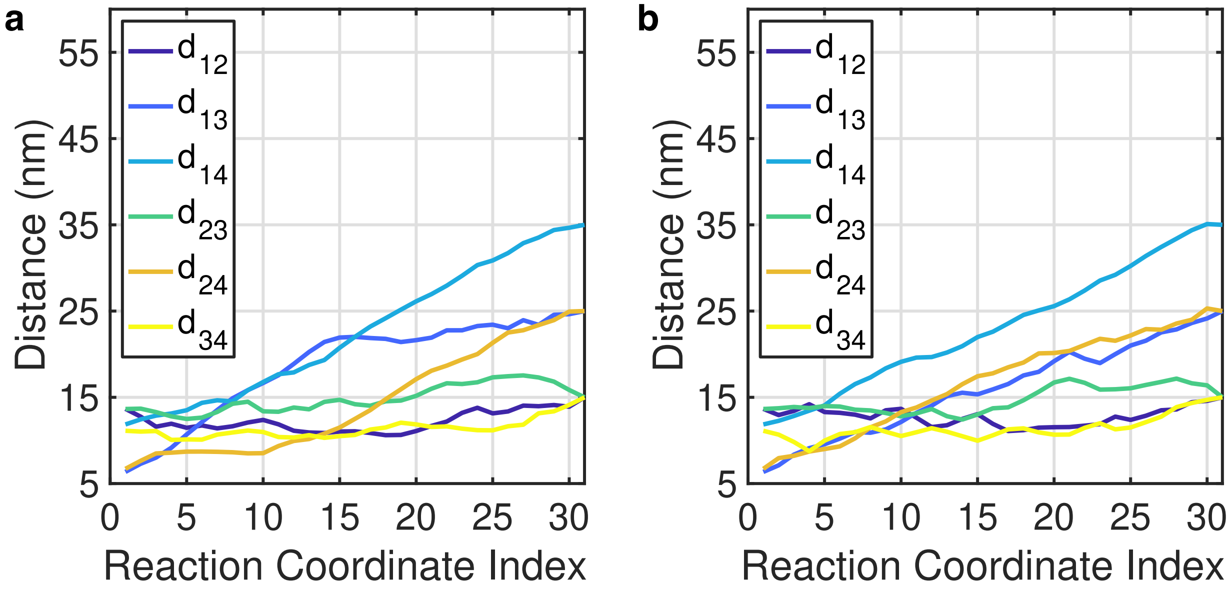

Figure S7: Variation of the inter-nucleosome distances along the sequential (a) and concerted (b) folding pathways shown in Fig. 4 of the main text.

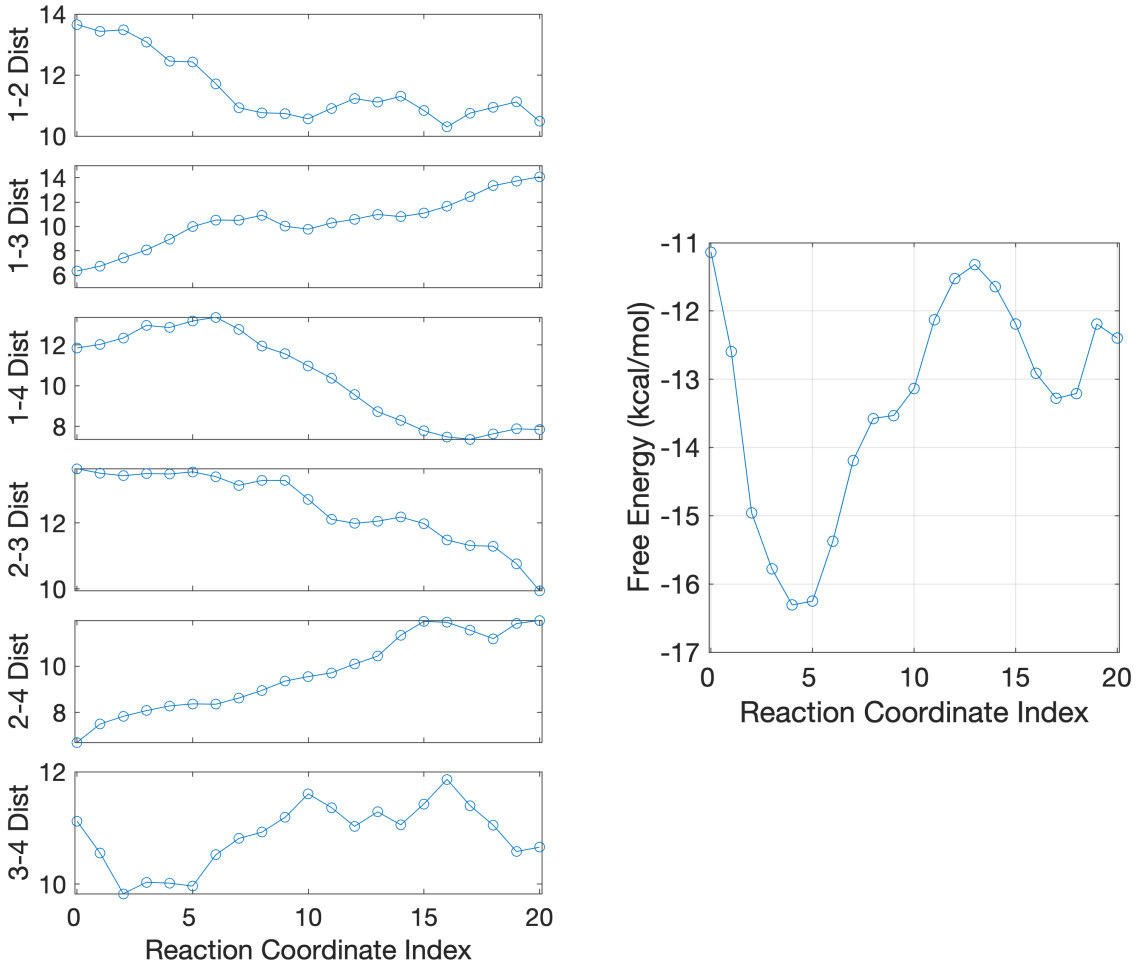

Figure S8: **Variation of the inter-nucleosome distances along a path that connects the anti-parallel structure (reaction coordinate 20) with the stacked zigzag structure (reaction coordinate 0).** 3D renderings of these two structures are provided in Fig. 2 of the main text. The path was again obtained from the finite temperature string method. The free energy profile along the path is shown on the right. The initial increase of 1-4 distance along the path indicates that the tetra-nucleosome configuration expands first to undo the inter-nucleosome contacts before further collapsing to the stacked zigzag structure. The expansion leads to the free energy barrier at bead 13. Therefore, the anti-parallel structure resembles kinetic traps rather than folding intermediates *en route* to the zigzag configuration. Interestingly, similar kinetics for the transition between the two structures was observed in our biased simulations as well (see Supplementary Movie 2).

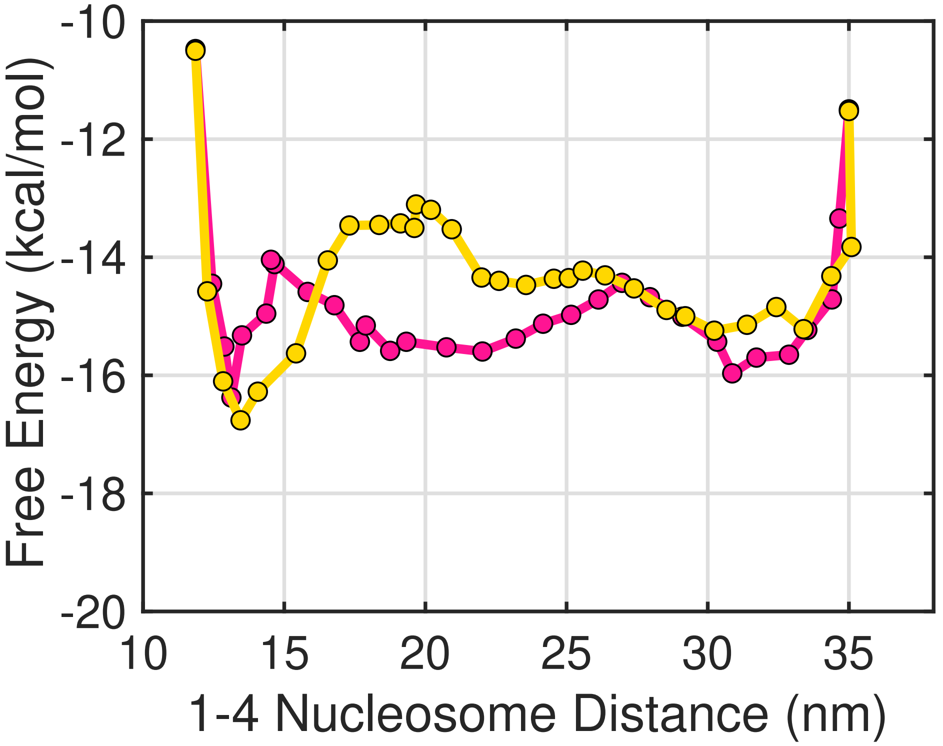

Figure S9: **Recalibrated free energy profiles as a function of the 1-4 nucleosome distance for the two pathways presented in Fig. 4 of the main text.** We subtracted the free energy with $-f\Delta z$ to mimic the impact of a pulling force. Δz is the difference in the 1-4 nucleosome distance from the free energy minimum (reaction coordinate index 3, and $\text{dist}_{14} = 12.8$ nm). $f = 4.5$ pN was estimated from prior single-molecule force spectroscopy experiments.[25,26] The emergence of an additional minimum at large inter-nucleosome distances (reaction coordinate index 28, and $\text{dist}_{14} = 33.4$ nm) with comparable stability to the original free energy minimum indicates the transition into more extended chromatin conformations. The extension per nucleosome between the open and closed chromatin state is 5.1 nm, which is in excellent agreement with the experimental value of 5 nm.[27] The nucleosome stacking energy per nucleosome, i.e., half of the free energy difference between the open state and the stacked structure, is 13.3 k_BT, which is also in good agreement with the experimental value of 14 k_BT.

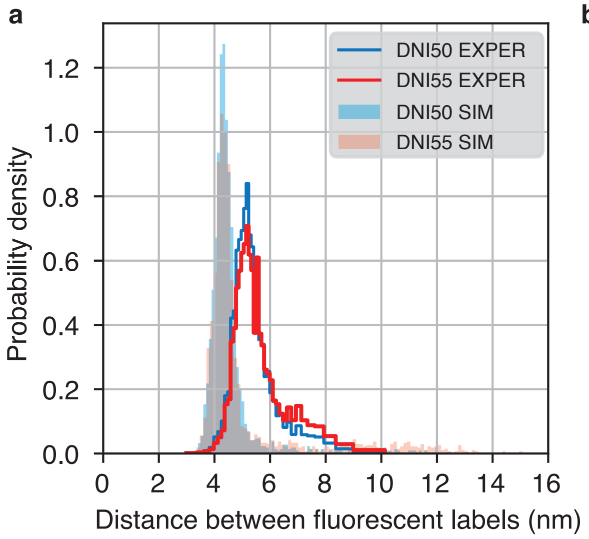

Figure S10: **The near-atomistic model reproduces the impact of linker DNA length on nucleosome unwrapping.** (a) Comparison between simulated (SIM) and experimental (EXPER) probability distribution of the donor-acceptor distance for dinucleosomes with 50 (DNI50) and 55 (DNI55) bp long linker DNA. (b) Comparison between experimental and simulated probability density distribution of the FRET efficiency. The magenta bar indicates the threshold efficiency ($E = 0.3$) used to defined the unwrapped state. See text *Section: Simulation details of dinucleosomes with different linker lengths* for further discussion.

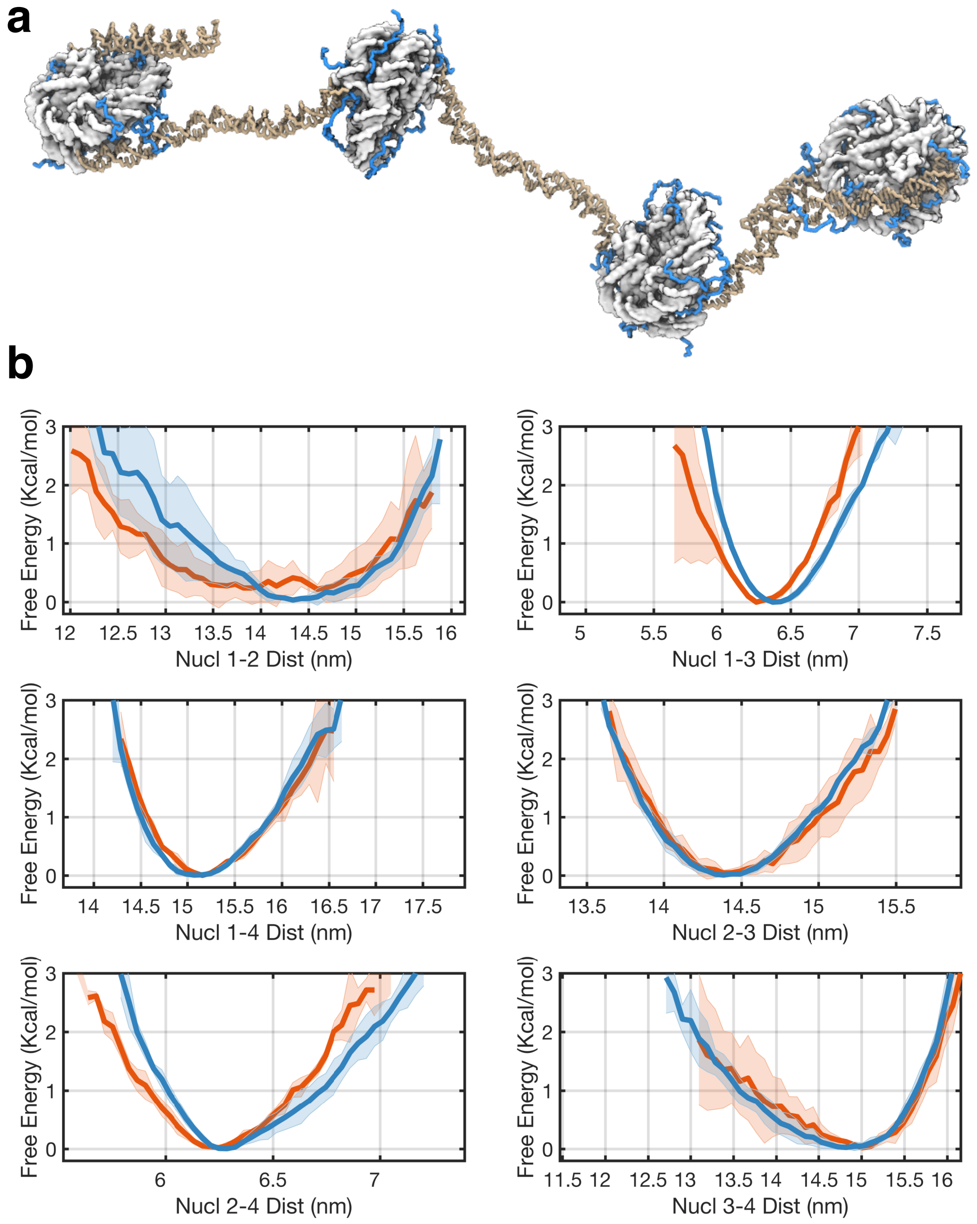

Figure S11: **Rigid body treatment of inner nucleosome core does not impact force field accuracy**. (a) An example structure of the tetra-nucleosome with the rigidified parts colored in white. Linker and outer layer DNA is shown in yellow. Disordered histone tails are shown in blue. (b) Comparison between the free energy profiles of various inter-nucleosome distances calculated with (blue) and without (red) rigid body dynamics. Shaded regions represent standard deviations of the mean estimated via block averaging. See text *Rigid body treatment* for simulation details.

Table S1: Trajectory length for simulations performed to calculate the free energy as a function of d_{13} using umbrella sampling. See text *Section:Free energy as a function of* d_{13} *calculated from umbrella sampling* for simulation details.

Umbrella Windows	Simulation Steps
d^o_{13} = 5nm	69246000
d^o_{13} = 8nm	63823000
d^o_{13} = 9nm	71969000
d^o_{13} = 10nm	70063000
d^o_{13} = 13nm	41941000
d^o_{13} = 15nm	60282000
d^o_{13} = 20nm	37501000
d^o_{13} = 24nm	60210000
d^o_{13} = 25nm	51107000
d^o_{13} = 8.5nm	70956000
d^o_{13} = 12.5nm	70022000
d^o_{13} = 17.5nm	52390000
d^o_{13} = 22.5nm	39321000

Table S2: Summary of umbrella simulations of the tetranucleosome with the SIRAH force field.[17]

Index	Umbrella center (rad)	Restraining constant (kcal/mol · rad^2)	Length (μs)
1	0.5	5.0	1.55
2	0.75	5.0	1.70
3	1.0	5.0	1.60
4	1.25	5.0	1.72
5	1.5	5.0	1.55
6	1.75	5.0	1.10
7	2.0	5.0	1.70
8	2.25	5.0	1.59
9	2.5	5.0	0.82
10	2.75	5.0	1.30
11	0.8	5.0	1.59
12	0.95	5.0	1.50
13	1.1	5.0	1.60
14	1.25	5.0	1.65
15	1.4	5.0	1.50
16	1.55	5.0	1.57
17	1.7	5.0	1.65
18	1.85	5.0	1.45
19	2.0	5.0	1.65
20	2.15	5.0	1.64
21	0.5	10.0	1.0
22	1.0	10.0	1.0

References

(1) Schalch, T.; Duda, S.; Sargent, D. F.; Richmond, T. J. X-ray structure of a tetranucleosome and its implications for the chromatin fibre. *Nature* **2005**, *436*, 138–141, DOI: 10.1038/nature03686.

(2) Collepardo-Guevara, R.; Portella, G.; Vendruscolo, M.; Frenkel, D.; Schlick, T.; Orozco, M. Chromatin unfolding by epigenetic modifications explained by dramatic impairment of internucleosome interactions: A multiscale computational study. *J. Am. Chem. Soc.* **2015**, *137*, 10205–10215, DOI: 10.1021/jacs.5b04086.

(3) Freeman, G. S.; Hinckley, D. M.; Lequieu, J. P.; Whitmer, J. K.; De Pablo, J. J. Coarse-grained modeling of DNA curvature. *J Chem Phys* **2014**, *141*, 165103, DOI: doi:http://dx.doi.org/10.1063/1.4897649.

(4) Clementi, C.; Nymeyer, H.; Onuchic, J. N. Topological and energetic factors: What determines the structural details of the transition state ensemble and 'en-route' intermediates for protein folding? An investigation for small globular proteins. *J. Mol. Biol.* **2000**, *298*, 937–953, DOI: 10.1006/jmbi.2000.3693.

(5) Noel, J. K.; Whitford, P. C.; Sanbonmatsu, K. Y.; Onuchic, J. N. SMOG@ctbp: Simplified deployment of structure-based models in GROMACS. *Nucleic Acids Res.* **2010**, *38*, 657–661, DOI: 10.1093/nar/gkq498.

(6) Noel, J. K.; Whitford, P. C.; Onuchic, J. N. The shadow map: A general contact definition for capturing the dynamics of biomolecular folding and function. *J. Phys. Chem. B* **2012**, *116*, 8692–8702, DOI: 10.1021/jp300852d.

(7) Noel, J. K.; Levi, M.; Raghunathan, M.; Lammert, H.; Hayes, R. L.; Onuchic, J. N.; Whitford, P. C. SMOG 2: A Versatile Software Package for Generating Structure-Based Models. *PLoS Comput. Biol.* **2016**, *12*, 1–14, DOI: 10.1371/journal.pcbi.1004794.

(8) Zhang, B.; Zheng, W.; Papoian, G. A.; Wolynes, P. G. Exploring the Free Energy Landscape of Nucleosomes. *J. Am. Chem. Soc.* **2016**, *138*, 8126–8133, DOI: 10.1021/jacs.6b02893.

(9) Kingma, D. P.; Ba, J. *Adam: A Method for Stochastic Optimization.* 3rd International Conference on Learning Representations, ICLR 2015, San Diego, CA, USA, May 7-9, 2015, Conference Track Proceedings. 2015.

(10) Vanden-Eijnden, E.; Venturoli, M. Revisiting the finite temperature string method for the calculation of reaction tubes and free energies. *J. Chem. Phys.* **2009**, *130*, 194103, DOI: 10.1063/1.3130083.

(11) Shirts, M. R.; Chodera, J. D. Statistically optimal analysis of samples from multiple equilibrium states. *J. Chem. Phys.* **2008**, *129*, 1–10, DOI: 10.1063/1.2978177.

(12) Ding, X.; Vilseck, J. Z.; Brooks, C. L. Fast Solver for Large Scale Multistate Bennett Acceptance Ratio Equations. *J. Chem. Theory Comput.* **2019**, *15*, 799–802, DOI: 10.1021/acs.jctc.8b01010.

(13) Buning, R.; Kropff, W.; Martens, K.; van Noort, J. spFRET reveals changes in nucleosome breathing by neighboring nucleosomes. *J. Phys. Condens. Matter* **2015**, *27*, 064103, DOI: 10.1088/0953-8984/27/6/064103.

(14) Lu, X.-J. 3DNA: a software package for the analysis, rebuilding and visualization of three-dimensional nucleic acid structures. *Nucleic Acids Research* **2003**, *31*, 5108–5121, DOI: 10.1093/nar/gkg680.

(15) Vasudevan, D.; Chua, E. Y.; Davey, C. A. Crystal Structures of Nucleosome Core Particles Containing the 601 Strong Positioning Sequence. *Journal of Molecular Biology* **2010**, *403*, 1–10, DOI: 10.1016/j.jmb.2010.08.039.

(16) Koopmans, W.; Buning, R.; Schmidt, T.; van Noort, J. spFRET Using Alternating Excitation and FCS Reveals Progressive DNA Unwrapping in Nucleosomes. *Biophysical Journal* **2009**, *97*, 195–204, DOI: 10.1016/j.bpj.2009.04.030.

(17) Machado, M. R.; Barrera, E. E.; Klein, F.; Sora, M.; Silva, S.; Pantano, S. The SIRAH 2.0 Force Field: Altius, Fortius, Citius. *Journal of Chemical Theory and Computation* **2019**, *15*, 2719–2733, DOI: 10.1021/acs.jctc.9b00006.

(18) Barrera, E. E.; Machado, M. R.; Pantano, S. Fat SIRAH: Coarse-Grained Phospholipids to Explore Membrane-Protein Dynamics. *J. Chem. Theory Comput.* **2019**, *15*, 5674–5688, DOI: 10.1021/acs.jctc.9b00435.

(19) Machado, M. R.; Barrera, E. E.; Klein, F.; Sónora, M.; Silva, S.; Pantano, S. The SIRAH 2.0 Force Field: Altius, Fortius, Citius. *J. Chem. Theory Comput.* **2019**, *15*, 2719–2733, DOI: 10.1021/acs.jctc.9b00006.

(20) Brandner, A.; Schüller, A.; Melo, F.; Pantano, S. Exploring DNA dynamics within oligonucleosomes with coarse-grained simulations: SIRAH force field extension for protein-DNA complexes. *Biochem. Biophys. Res. Commun.* **2018**, *498*, 319–326, DOI: 10.1016/j.bbrc.2017.09.086.

(21) Bussi, G.; Donadio, D.; Parrinello, M. Canonical sampling through velocity rescaling. *The Journal of Chemical Physics* **2007**, *126*, 014101, DOI: 10.1063/1.2408420.

(22) Parrinello, M.; Rahman, A. Polymorphic transitions in single crystals: A new molecular dynamics method. *Journal of Applied Physics* **1981**, *52*, 7182–7190, DOI: 10.1063/1.328693.

(23) Essmann, U.; Perera, L.; Berkowitz, M. L.; Darden, T.; Lee, H.; Pedersen, L. G. A smooth particle mesh Ewald method. *The Journal of Chemical Physics* **1995**, *103*, 8577–8593, DOI: 10.1063/1.470117.

(24) Abraham, M. J.; Murtola, T.; Schulz, R.; Pll, S.; Smith, J. C.; Hess, B.; Lindahl, E. GROMACS: High performance molecular simulations through multi-level parallelism from laptops to supercomputers. *SoftwareX* **2015**, *1-2*, 19–25, DOI: 10.1016/j.softx.2015.06.001.

(25) Kruithof, M.; Chien, F. T.; Routh, A.; Logie, C.; Rhodes, D.; Van Noort, J. Single-molecule force spectroscopy reveals a highly compliant helical folding for the 30-nm chromatin fiber. *Nat. Struct. Mol. Biol.* **2009**, *16*, 534–540, DOI: 10.1038/nsmb.1590.

(26) de Jong, B. E.; Brouwer, T. B.; Kaczmarczyk, A.; Visscher, B.; van Noort, J. Rigid Basepair Monte Carlo Simulations of One-Start and Two-Start Chromatin Fiber Unfolding by Force. *Biophys. J.* **2018**, *115*, 1848–1859, DOI: 10.1016/j.bpj.2018.10.007.

(27) Norouzi, D.; Zhurkin, V. B. Dynamics of Chromatin Fibers: Comparison of Monte Carlo Simulations with Force Spectroscopy. *Biophys. J.* **2018**, *115*, 1644–1655, DOI: 10.1016/j.bpj.2018.06.032.
