# Supplementary material for: Stability and folding pathways of tetra-nucleosome from six-dimensional free energy surface
Source: Nat Commun. 2021 Feb 17;12:1091. doi: 10.1038/s41467-021-21377-z (PMC7889939; doi:10.1038/s41467-021-21377-z)
Supplement: Supplementary file 3 — Description of Additional Supplementary Files [file 41467_2021_21377_MOESM3_ESM.pdf]

Description of Additional Supplementary Files

Supplementary Movie 1
Representative tetra-nucleosome configurations from K-means clustering. For clarity, only the DNA molecule, the color of which varies from green to white, is shown. The N-terminal tails of histone H3 and H4 protein are drawn in purple and orange, respectively.

Supplementary Movie 2
Illustration of the concerted folding pathway obtained from dynamical simulations.

Supplementary Movie 3
Illustration of the sequential folding pathway obtained from dynamical simulations.

Supplementary Movie 4
Illustration of the concerted folding pathway obtained from string method calculations.

Supplementary Movie 5
Illustration of the sequential folding pathway obtained from string method calculations.
